# Supplementary material for: Markov reads Puškin, again: A statistical journey into the poetic world of Evgenij Onegin
Source: PLoS One. 2026 Jun 4;21(6):e0350827. doi: 10.1371/journal.pone.0350827 (PMC13235941; doi:10.1371/journal.pone.0350827)
Supplement: S1 Appendix — Describes the rules adopted for classifying characters as vowels or consonants in both the Russian original and the Italian translation. (PDF) [file pone.0350827.s001.pdf]

S1 Appendix. Encoding rules for V/C classification

Each line of text was encoded as a binary sequence of vowels (V) and consonants (C). The Russian encoding followed post-1918 orthographic conventions. Following Markov's approach, the semivowel й was treated as a vowel, and non-alphabetic characters (punctuation, digits, whitespace) were excluded. The soft sign (ь) and hard sign (ъ), while phonologically relevant, were retained for character counts but excluded from V/C classification. Unlike Markov, we applied a standard vowel/consonant classification to Latin characters; thus, foreign expressions in Latin script—mostly in French, but also in English, Italian, or German—were preserved. The same procedure was applied to the Italian translation, with classification based on orthographic structure, including accented and diacritic forms. Foreign insertions (e.g., Gallicisms, Anglicisms) were encoded accordingly, without normalization.

Characters were preserved in their original case, but converted to lowercase during encoding. The final classification scheme was as follows:

- **Vowels (V):**
  - *Cyrillic:* а, е, ё, и, о, у, ы, э, ю, я, й
  - *Latin:* a, e, i, o, u,
    accented: á, é, í, ó, ú, à, è, ì, ò, ù
    circumflex: â, ê, î, ô, û
    umlaut: ä, ë, ï, ö, ü
- **Consonants (C):**
  - *Cyrillic:* б, в, г, д, ж, з, к, л, м, н, п, р, с, т, ф, х, ц, ч, ш, щ
  - *Latin:* b, c, d, f, g, h, j, k, l, m, n, p, q, r, s, t, v, w, x, y, z
    additional: č, š, ž, ỳ
- **Excluded (not encoded):** The soft sign (ь), hard sign (ъ), digits, punctuation (including quotation marks and apostrophes), and whitespace were removed prior to encoding and did not contribute to sequence generation.
