# Supplementary material for: Markov reads Puškin, again: A statistical journey into the poetic world of Evgenij Onegin
Source: PLoS One. 2026 Jun 4;21(6):e0350827. doi: 10.1371/journal.pone.0350827 (PMC13235941; doi:10.1371/journal.pone.0350827)
Supplement: S2 Appendix — Includes Table A1 reporting the estimated linear trends in MD under alternative block length configurations. (PDF) [file pone.0350827.s002.pdf]

S2 Appendix. Sensitivity of block length selection

To assess the robustness of the block-based analysis, the Russian corpus was reprocessed using an alternative block length of 8,000 characters (main analysis: 10,000). For each configuration, a linear trend was estimated by regressing the blockwise values of $\mathrm{MD} = 1 - cf_{\text{complex}}$ against blockwise position within the text (scaled to the interval $[0, 1]$). The estimated slopes were comparable in sign and magnitude (Table A1), indicating that the qualitative trend is not sensitive to the specific choice of block length.

Table A1. Sensitivity check for block length selection in the Russian corpus. Linear trends were estimated from $\mathrm{MD} \sim t$, where t denotes blockwise position in $[0, 1]$.

Block length (chars)	Slope $\hat{\beta}_t$	Std. Error
8,000	-0.02262	0.00688
10,000	-0.02205	0.00776
