# Supplementary material for: Markov reads Puškin, again: A statistical journey into the poetic world of Evgenij Onegin
Source: PLoS One. 2026 Jun 4;21(6):e0350827. doi: 10.1371/journal.pone.0350827 (PMC13235941; doi:10.1371/journal.pone.0350827)
Supplement: S3 Appendix — Includes Table B1 (lemma corrections for the probe “вст”) and Table B2 (mapping of stems to thematic labels used in probe classification). (PDF) [file pone.0350827.s003.pdf]

S3 Appendix. Lemmatization and thematic mapping for phonological probes

Manual corrections for lemmas containing the phonological probe ”вст”

Table B1 documents the single-word occurrences of the phonological probe ”вст” in *Evgenij Onegin* for which UPOS corrections were needed. These corrections were made manually based on morphological analysis, syntactic context, and direct inspection of the poetic text. Special attention was paid to poetic and metrically truncated forms that are known to often confound automatic lemmatization systems.

Table B1. Lemmatized single-word matches of the probe ”вст”. They are grouped by lexical stem and annotated with corrected UPOS tags and English translations. All forms were manually reviewed to account for poetic variants and tagging errors.

lemma	stem	UPOS	translation
встретить	встр	VERB	to meet
встретиться	встр	VERB	to meet (reflexive)
встретя	встр	VERB	having met
встреча	встр	NOUN	meeting
встречу	встр	NOUN	meeting (accusative singular)
встречный	встр	ADJ	opposing / counter
вступать	вступ	VERB	to enter / to join
вступить	вступ	VERB	to enter / to join
вступленье	вступ	NOUN	entrance (poetic form)
вступление	вступ	NOUN	entrance
предчувствие	чувств	NOUN	foreboding / premonition
чувство	чувств	NOUN	feeling / emotion
чувствоваться	чувств	VERB	to be felt
чувствовать	чувств	VERB	to feel
чувствительный	чувств	ADJ	sensitive

Thematic mapping of ”вст” stems

To support root-based thematic classification, we performed stemming of lemmatized forms using the SnowballC algorithm. Although not used directly for tagging, stem clusters helped validate the coherence of lexical groupings, as summarized in Table B2.

Table B2. Mapping of phonological stems to thematic labels used in the recomputation of blockwise frequency profiles. Comments clarify the semantic rationale behind each label.

stem	thematic label	comment
встр	encounter	core lexeme cluster expressing meeting or confrontation
вступ	encounter	semantically related to entry/initiation themes
здравст	encounter	appears in greetings, often initiating contact
бесчувст	emotion	negative affect: coldness or lack of empathy
вста	emotion	conveys emergence, transition, or emotional awakening
девств	emotion	used metaphorically in purity or vulnerability
предчувств	emotion	literally ”pre-feeling”, linked to anticipation
чувств	emotion	expresses affective states or sensitivity
